# 

**Published:** 1862-03

**Authors:** 


### THE
### DENTAL REGISTER
### OF THE WEST.

EDITED BY

J. TAFT AND GEO. WATT.

SMITHSONIAN INSTITUTION

MARCH,–1862.

MONTHLY:
Three Dollars per Annum, in advance.

CINCINNATI:
J. TAFT, PUBLISHER.

J. TAFT, DENTIST, NO. 56 WEST FOURTH STREET.

